# Specific foods can reduce symptoms of irritable bowel syndrome and functional constipation: a review

**DOI:** 10.1186/s13030-019-0152-5

**Published:** 2019-05-08

**Authors:** Yohei Okawa, Shin Fukudo, Hiromi Sanada

**Affiliations:** 1 Graduate School of Medicine Director, Global Nursing Research Center, University of Tokyo, 7-3-1 Hongo, Bunkyo-ku, Tokyo, 113-0033 Japan; 2 Department of Behavioral Medicine, Tohoku University Graduate School of Medicine, Sendai, Japan

**Keywords:** Irritable bowel syndrome IBS, Functional constipation FC, Functional gastrointestinal disorders FGIDs, Fermentable oligosaccharides Disaccharides Monosaccharaides And polyols FODMAP, Functional dyspepsia FD

## Abstract

**Background:**

Functional gastrointestinal disorders (FGIDs) are groups of disorders involving digestive symptoms that chronically persist despite the absence of organic abnormalities in the gastrointestinal tract. Representative FGIDs include functional dyspepsia (FD), irritable bowel syndrome (IBS), and functional constipation (FC). In particular, IBS is a disease representative of FGIDs in which abdominal pain and discomfort associated with abnormal bowel movements chronically persist and recur. These symptoms are known to be related to lifestyle habits, such as meals and stress. In recent years, according to advances in dietary therapy for IBS and FC, specific foods have been shown to alter these symptoms. In IBS, bowel movement abnormalities and abdominal discomfort have been reported to be reduced when patients eat these specific foods.

**Main topic:**

Several studies suggest that individuals with certain attitudes toward eating or with preferences for fatty food, fast food, junk snack food, fried food, and hot/spicy food showed a higher prevalence of gastrointestinal (GI) symptoms. Those who are cognizant of nutritional balance or healthy food intake have a lower prevalence of GI symptoms. Thus, eating specific foods with higher dietary fiber and low FODMAP (fermentable oligosaccharides, disaccharides, monosaccharaides, and polyols) is effective for relief from some GI symptoms.

First, two kinds of dietary fibers are found in foods: water-soluble dietary fiber and insoluble dietary fiber. Enduring misconceptions about the physical effects of fiber in the gut have led to misunderstandings about the health benefits attributable to insoluble and soluble fiber. Previous reviews suggest that health benefits have been shown in regard to fiber, and reproducible evidence of clinical efficacy has been published.

Second, the ingestion of certain carbohydrates causes gastrointestinal symptoms. Foods rich in fermentable oligosaccharides, disaccharides, monosaccharaides, and polyols (collectively known as FODMAP) have been shown to cause abdominal pain and abdominal discomfort in westerners with IBS.

**Conclusion:**

Dietary therapy for FGIDs should include specific foods that have been scientifically proven to be effective for managing symptoms of irritable bowel syndrome and functional constipation.

## Background

### Definition of functional digestive tract disease

Functional gastrointestinal disorders (FGIDs) are groups of disorders with digestive symptoms that chronically persist despite the absence of organic abnormalities in the gastrointestinal tract. Many patients with FGIDs have functional psychosomatic symptoms, and the association of the gastrointestinal tract with brain function due to the cerebral-intestinal correlation has been suggested [1] [2] [3]. Representative FGIDs include functional dyspepsia (FD), irritable bowel syndrome (IBS), and functional constipation (FC). In particular, IBS is a representative FGID in which abdominal pain and discomfort associated with an abnormal bowel movement chronically persist and recur [1] [2] [4]. The diagnostic criteria of Rome IV [1], which is an international diagnostic criterion, is used for the diagnosis of IBS [2]. This diagnostic criteria is as follows: “abdominal pain that persists at least 3 days or more per month in the last 3 months, and its symptoms are improved by ① defecation, ② a change in the defecation frequency, ③a change in the number of items 1, 2, and 3.” IBS is diagnosed as having symptoms for more than 6 months and is classified into the following four subtypes on the basis of the frequency of the Bristol stool form scale (BSFS) of bowel movement [5]: IBS constipation type (IBS-C), diarrhea type (IBS-D), mixed type (IBS-M), and nonclassifiable type (IBS-U). Among those who meet the IBS diagnostic criteria, IBS-C is defined as being IBS-M if the frequency of diarrhea is as low as < 25%, and IBS-M when the frequency of diarrhea and loose stools occur in excess of 25% in addition to constipation [6] [7]. IBS-C is accompanied by abdominal pain and discomfort that occur in relation to bowel movements [8] [7]. The prevalence of IBS-C is high in Japan. In a survey of 30,000 adults aged 20 to 79 years, 4942 had IBS (prevalence rate 16.5% [male 15.5%, female 17.4%]); among those with IBS, IBS-C was slightly less frequent (2.8%: 1.5% male, 4.0% female) compared with other subtypes (IBS-D 4.5%, IBS-M 8.3%). The most frequently occurring symptoms of IBS-C are abdominal fullness (80.1%), followed by intestinal gas overstimulation (71.3%), abdominal discomfort (64.3%), and abdominal pain (29.1%). More than 80% of patients have reported a feeling of abdominal bloating as a symptom of IBS-C, which has been reported to be correlated with anxiety in daily life [9].

A person with FC who does not meet the criteria of IBS-C based on the Rome III diagnostic criteria presents with the following characteristics: more than 25% of the usual defecation, more than 25% hard stool, a sense of obstruction in the rectum of 25% or more, manual defecation assistance of 25% or more, and defecation frequency of < 3 times per week [6]. The diagnostic criteria for FC consist of symptoms that suggest a failure of gastrointestinal transit ability and fecal efflux. Among these items, only “abdominal pain” or “abdominal discomfort” correspond to IBS, whereas FC does not exhibit abdominal symptoms [6].

## Main topic

### What are the lifestyle-related causes of IBS symptoms?

Factors associated with the onset of IBS can be genetic, environmental, related to infection, inflammation, intestinal bacteria, and stress [6]. Stress, as a psychosocial factor, is involved to a large extent, as stress induces hyperalgesia and gastrointestinal movement disorder. Furthermore, lifestyle factors, such as diet, exercise, and sleep may cause symptoms in IBS patients [9]. Regarding lifestyle, food consumption is an indispensable act in daily life. Certain foods may reduce digestive symptoms by altering colonic transit. Thus, it is important to examine how certain types of food can reduce digestive symptoms.

### Characteristics of the pathogenesis of IBS and FC (Fig. 1)

In understanding the mechanism of the generation of IBS, a previous study suggests brain-gut interaction [10]. Brain-gut interaction has been shown to be related to digestive tract movement, visceral hypersensitivity, and psychological abnormalities; therefore, these interactions are an important part of the pathophysiology of IBS [11]. With IBS in particular, when a stretching stimulus is added to the large intestine, a decrease in gastrointestinal pain threshold is observed, and healthy individuals become more aware of gastrointestinal perception compared to stimulus-induced awareness of gastrointestinal perception [12]. There is some allodynia that causes abdominal pain and even physiological stimulation and hyperalgesia that reacts excessively when a noxious stimulus is added. In addition, it has been reported that the discomfort threshold of IBS patients is lower than that of healthy subjects for electrical stimulation [13] [14]. However, the relation between IBS symptoms and sensory hypersensitivity reactions has not yet been clarified. Furthermore, regarding the occurrence of IBS symptoms, brain function has been suggested as a symptom related to visceral perception in the gastrointestinal tract [2] [3] [11] [14].

**Fig. 1:**
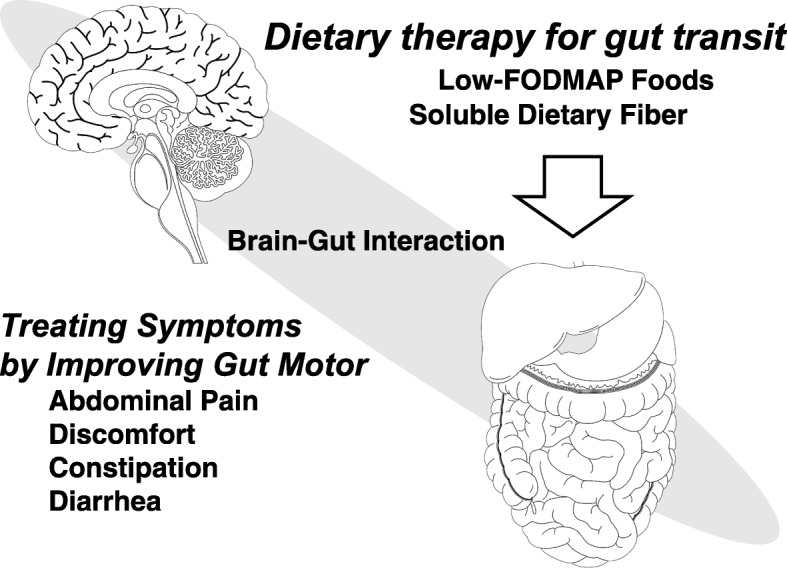
Brain-gut interaction were related to digestive tract movement, visceral hypersensitivity, and psychological abnormalities

In the pathogenesis in symptoms, first, the gastrointestinal tract is stimulated. This simulation is transmitted to the sensory neurons of the spinal nerve as a nociceptive stimulus signal. Then, the signal is transferred to the visceral sensory neurons existing in the dorsal horn of the spinal cord via the dorsal root of the spinal cord, and the stimulus is conveyed. Furthermore, the signal travels up to the thalamus and is then projected to the insula, the anterior cingulate gyrus, and the prefrontal cortex. From there, the visceral perception is generated [9]. Hyperesthesia has been shown to occur when the intensity of this stimulus is strong or when the threshold of visceral perception for stimulation in the gastrointestinal tract is low [3] [11]. Especially in the case of IBS, the perceptual threshold for the gastrointestinal tract is suggested to decrease, and the perceptual sensitivity tends to be higher than in healthy subjects [9].

With IBS, visceral hypersensitivity movement abnormalities in the gastrointestinal tract have been shown to be associated with abdominal pain and discomfort [2] [4]. Previous studies have shown that gastrointestinal transit time (colonic transit time) is associated with abdominal pain and bloating in IBS-C [15]. In addition, the frequency of bowel movements and the shape of the stool were negatively correlated with the gastrointestinal transit time; thus, it is important to evaluate the transit time in the gastrointestinal tract of patients with IBS-C constipation symptoms [16]. Treatment for visceral hypersensitivity and gastrointestinal motility abnormalities that cause abdominal pain and discomfort in IBS is very important, as described above; however, treatment for the symptoms themselves have not been established yet, and an effective treatment method should be developed. We suggest that the occurrence of symptoms is related with lifestyle habits. For example, certain foods may be related to the occurrence of these symptoms. Further, as a clinical evaluation method of colorectal function in FGIDs, measurements of the colonic transit time using an abdominal X-ray opacity marker have been performed [17] [18]. Abdominal X-ray imaging makes it possible to evaluate gastrointestinal function based on the number of residual markers in the gastrointestinal tract and the residual site [19] [20]. In addition, colonic transit time is classified as normal colonic transit (< 3 days) (normal transit constipation) and delayed colon transit (≥3 days) (slow transit constipation), which has been reported to be associated with pelvic floor dysfunction [19]. In a study of the colonic transit time in IBS patients, 287 cases (80%) were normal, 19 cases (5%) were the delayed type, and 53 cases (15%) were the shortened type (accelerated) [15]. Furthermore, when comparing the gastrointestinal transit times for each subtype of IBS (IBS-C, IBS-D, and IBS-M) according to the Rome III diagnostic criteria, 15% of cases in the IBS-C group exhibited delayed colonic transit, and only 36% of cases in the IBS-Ds exhibited accelerated gastrointestinal transit (accelerated); furthermore, many IBS patients have been reported to exhibit normal transit times [15]. The normal type overlaps each IBS subtype, and the delayed type accounts for 15% of IBS-C cases, suggesting that gastrointestinal motility is reduced compared with the other subtypes [15]. Furthermore, previous studies have compared transit time in the gastrointestinal tract between IBS-C and FC, and the difference in colonic transit time between these conditions was not significant [8]. In contrast, IBS-C involves significantly higher abdominal distension (bloating) and pain compared to FC, and these IBS symptoms have been shown to be correlated with colonic transit time [16]. Therefore, these symptoms are related to altered colonic transit. It is important to ascertain the cause of the alteration in colonic transit in IBS and FC.

### Association with foods and gastrointestinal symptoms

Previous studies have shown that abdominal pain and discomfort associated with IBS are affected by certain foods, i.e., symptoms are exacerbated by the ingestion of specific foods [14] [21] [22] [23]. Another previous study also suggested that food-related GI symptoms, particularly gas and abdominal pain, are of major importance in patients with IBS. Foods that are rich in carbohydrates, foods that contain dietary fiber, starch, lactose, fructose and sorbitol, and fatty foods are considered by many patients to produce GI symptoms, along with food agents such as coffee, alcohol and spices [24]. Although the majority of IBS patients experience severe GI symptoms after meals and the ingestion of individual foods, most of these patients are of normal weight or are overweight. Being female sex and having anxiety are two factors that appear to predict a high degree of food-related subjective GI symptoms in IBS [24]. Thus, in patients with IBS, the postprandial worsening of symptoms, as well as adverse reactions to one or more foods, are common.

### Can dietary fiber improve symptoms in IBS-C and FC?

The following two types of dietary fiber are found in food: water-soluble dietary fiber and insoluble dietary fiber. Enduring misconceptions about the physical effects of fiber in the gut have led to misunderstandings about the health benefits that are attributable to insoluble and soluble fiber. A previous review described the health benefits of dietary fiber based on reproducible evidence of clinical efficacy [25]. In the large bowel, there are two mechanisms that exert a laxative effect. Large/coarse insoluble fiber particles (e.g., wheat bran) mechanically irritate the gut mucosa, stimulating the secretion of water and mucous, and the high water-holding capacity of gel-forming soluble fiber (e.g., psyllium) resists dehydration. This mechanism requires that the fiber resist fermentation and remain relatively intact throughout the large bowel (the fiber must be present in stool); this mechanism leads to an increase in the water content of the stool, thereby resulting in a bulky/soft/easy-to-pass stool. Soluble fermentable fibers (e.g., inulin, fructooligosaccharide, and wheat dextrin) do not exert a laxative effect, and certain fibers can be constipating (e.g., wheat dextrin and fine/smooth insoluble wheat bran particles). When making recommendations for a fiber supplement, it is essential to recognize which fibers possess the physical characteristics required to provide a beneficial health effect and which fiber supplements are supported by reproducible, rigorous evidence of one or more clinically relevant studies reporting meaningful health benefits [25].

These considerations are important, as previous clinical studies have reported that water-soluble dietary fiber improves constipation symptoms [26] [27]. In a study comparing the effects of water-soluble dietary fiber and insoluble dietary fiber on IBS symptoms, the former resulted in a significant improvement in IBS symptoms, whereas the latter showed no improvement effects and even exacerbated symptoms in some cases [27] [26]. In particular, the effectiveness of psyllium (*Psyllium*) as a dietary fiber for the treatment of IBS symptoms has been demonstrated. Psyllium is a powder form of plants of the *Psyllium* species, and this powder contains many dietary fibers [23]. In a previous study on IBS, psyllium intake for 12 weeks was found to significantly improve IBS symptoms compared with other dietary fiber brands and a placebo [27]. In addition, the results of a randomized controlled trial involving patients with FC showed that the amount of complete natural defecation and value of BSFS significantly increased due to the intake of psyllium for 4 weeks, which significantly reduced straining and abdominal distention (bloating), suggesting that psyllium intake is effective for treating FC as well as IBS [28].

### Can a low-FODMAP diet improve symptoms in westerners with FGIDs?

Recently, the ingestion of certain carbohydrates has been shown to cause symptoms in people with FGIDs. Foods that are rich in fermentable oligosaccharides, disaccharides, monosaccharaides, and polyols (collectively known as FODMAP) have been shown to cause abdominal pain and abdominal discomfort in westerners with IBS [21] [22] [29] [30] [31] [23]. A previous review article reported that FODMAP-rich foods include chickpeas, lentils, and clingstone fruits, such as peaches and apricots, whereas low-FODMAP foods include kiwifruit, blueberry, grapefruit, and honeydew melon, among others [23]. Oligosaccharides and polyols, such as lactose, fructose, fructan, galactan, and others, that contain FODMAP are scarcely absorbed in the small intestine, and fermentation is subsequently promoted in the large intestine. Fermentation of these indigestible carbohydrates produces short-chain fatty acids in the large intestine. Short chain fatty acid is a generic term for acetic acid, propionic acid, butyric acid, isobutyric acid, lactic acid, and succinic acid, which are produced in the course of the fermentation of carbohydrates by intestinal bacteria such as *Lactobacillus* and *Veillonella*, which are increased in patients with IBS compared with healthy subjects. These bacteria have been suggested to produce short-chain fatty acids, resulting in abdominal pain and discomfort [23].

Low-FODMAP diets have been shown to be effective in several clinical trials [22] [21] [31]. A randomized controlled trial comparing a low-FODMAP diet with the conventional Australian diet showed that the low-FODMAP diet significantly improved the symptoms of IBS compared with the conventional Australian diet [21]. In a randomized controlled trial comparing a low-FODMAP diet with a high-FODMAP diet, the intake of low-FODMAP meals significantly reduced abdominal pain intensity, frequency, and IBS severity in individuals with IBS [31]. Furthermore, a previous review of four clinical studies on low-FODMAP diets, although the diet contents, observation period, and evaluation items differed among the studies, demonstrated the effectiveness of a low-FODMAP diet against IBS symptoms [22]. However, these studies only involved Westerners with IBS, and Japanese people and Westerners eat different foods. Previous studies have not sufficiently demonstrated the effect of a low-FODMAP diet for Japanese people. It remains unclear whether such diets are effective for Japanese people.

### What specific foods can reduce symptoms in Japanese people with IBS?

In Japan, a pervious study reported a relation between eating certain foods and FGID symptoms in the general Japanese population [32]. An evaluation of eating attitudes or food preference suggested that there was a higher prevalence of gastrointestinal (GI) symptoms among those who prefer fatty food, fast food, junk snack food, fried food, and hot/spicy food. In contrast, there was a lower prevalence of GI symptoms among those who were mindful of nutritional balance and ate healthy foods. Furthermore, wellness sensations (good eating, good bowel movement, and good sleep) were correlated with each other; i.e., those who have sleep impairments have a higher prevalence of impairments in terms of eating and bowel movements.

In addition, a previous study reported the association of the microbiota composition with habitual diet in Japanese people [33]. The number of microbiota was shown to be related to habitual diet. Bifidobacterium was correlated with iron intake, and the Clostridium cluster was negatively correlated with intakes of cholesterol and eggs. These findings suggested that dietary habits may strongly affect the abundance of *Bifidobacterium*, *Bacteroides,* and *Clostridium* in the gut microbiota of young Japanese women. However, it is unclear whether there is an association between the gut microbiota and digestion symptoms. GI symptoms in Japanese individuals are related to certain foods.

## Conclusion

We reported that bowel movement abnormalities and abdominal discomfort associated with FGIDs are related to specific foods, including dietary fiber and FODMAP-rich foods. As a dietary therapy, the specific foods that can effectively improve symptoms remain to be elucidated. In recent years, the effects of dietary fiber have been demonstrated [28]. Fiber components that exacerbate gastrointestinal symptoms are being elucidated. In addition, FODMAP-rich foods have been shown to be related to the onset and exacerbation of digestive symptoms in IBS [21] [34]. Regarding dietary therapy for treating FGIDs, there are specific foods that have been shown to be effective and foods for which there is insufficient evidence; it may be possible to improve symptoms if these foods are ingested in a balanced manner.

## Abbreviations

FC: Functional constipation
FGIDs: Functional gastrointestinal disorders
FODMAP: Fermentable oligosaccharides, disaccharides, monosaccharaides, and polyols
IBS: Irritable bowel syndrome
